# 741. Assessing the Concordance of MRSA Carriage Screening with MRSA Infections

**DOI:** 10.1093/ofid/ofad500.802

**Published:** 2023-11-27

**Authors:** Jonathan Mannheim, Madan Kumar, Palak Bhagat, Allison Nelson

**Affiliations:** University of Chicago Medicine, Chicago, Illinois; University of Chicago Medicine, Chicago, Illinois; University of Chicago Comer Children's Hospital, Chicago, Illinois; University of Chicago Comer Children's Hospital, Chicago, Illinois

## Abstract

**Background:**

There is a lack of research on MRSA surveillance in children. This study focused on children with confirmed MRSA infections to determine the utility of MRSA screens in guiding empiric anti-MRSA treatment for children without history of MRSA infection. We examined the concordance of screens to assess for differences by infection type, and used statistical analysis to determine significant contributors to concordance.

**Methods:**

Pediatric hospital patients admitted from 2002-2022 were included. Subjects had MRSA infections subsequent to a MRSA surveillance screen performed in the preceding year. Statistical analysis, namely multivariable and binomial regression, identified associations between MRSA screens and infections. Number needed to treat analysis calculated the utility of re-screening.

**Results:**

Among 246 subjects, 39.0% had concordant screens. 151 (61.4%) screens were obtained in the two weeks preceding infection. Sensitivity for bacteremia was 50.0% (n=42), for ET/respiratory 44.4% (n=81), for SSTI 29.4% (n=102). For children under 6 months of age, sensitivity was 35.9% (n=78). Multivariable analysis significantly associated days since MRSA screening and decreasing likelihood of concordance. Binomial regression modeled the probability of concordance to drop below 50.0% for all infections after four days, after six days for bacteremia specifically, and twelve days for ET/respiratory infections.

Probability of Concordance Over Time

**Figure:**
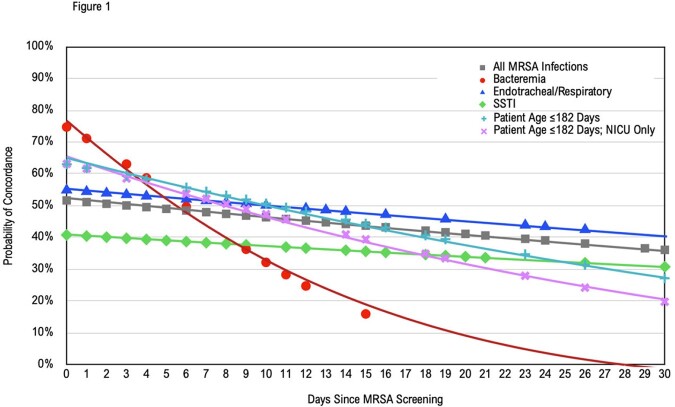

The probability of MRSA screening concordance over time based upon binomial prediction modeling. Each line represents the probability of concordance on a given day subsequent to MRSA screening.

Number Needed to Treat (test) Analysis

**Figure:**
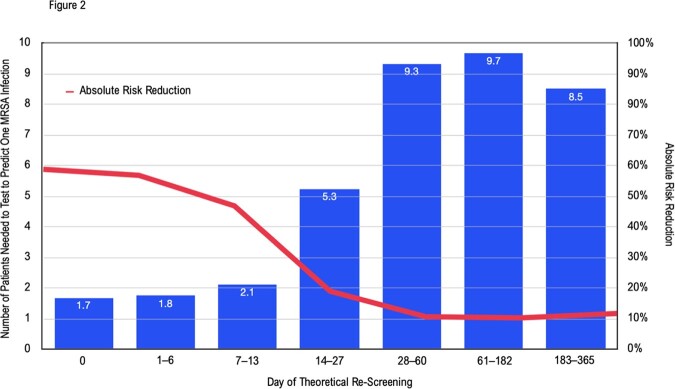

A theoretical control arm of patients receiving no re-screening is compared to a theoretical treatment arm receiving re-screening. Each column represents the number needed to re-screen (for a given number of days) to predict one MRSA infection. Absolute risk reduction is overlaid.

**Figure:**
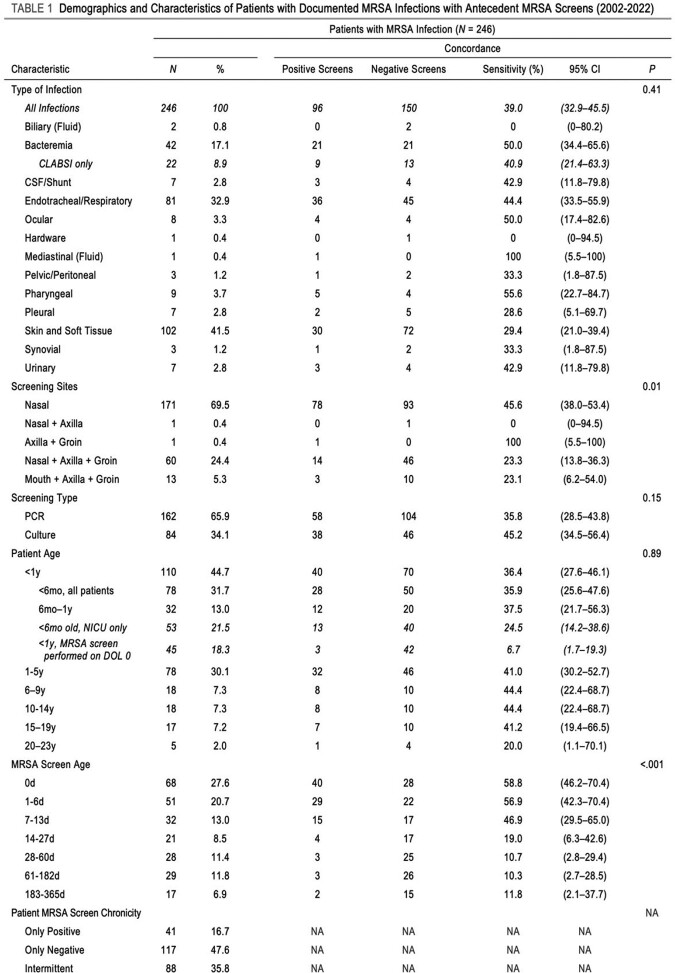

**Conclusion:**

The concordance of screens was far lower than NPV found in past studies. Bacteremia concorded at 50.0%, ET/respiratory infections 44.4%, SSTI 29.4%. The number of days elapsed since MRSA screening proved the only significant covariate. When that covariate modeled the probability of screening concordance with infection, 7 days represented when the probability of concordance fell to or dropped below 50.0% for infection types. Only patients with a known MRSA infection were included in our study, and concordance of screens was far lower than NPV found in past studies; previous results may have been impacted by low prevalence to inform high NPV. We suggest that negative MRSA screens should not invalidate reasonable suspicion for MRSA infection in patients with high pre-test probabilities for infection. Re-screening patients may optimize concordance.

**Disclosures:**

**All Authors**: No reported disclosures

